# 

**Published:** 1904-02-06

**Authors:** 


### Publications.

Just Published. 8vo. with Photographic reproductions, 3/- net.

**THE PHYSIOGNOMY OF MENTAL DISEASES AND DEGENERACY.** By JAMES SHAW, M.D, formerly Med. Supt. and Co-Licensee Haydock Lodge Asylum. Reprinted, with alterations and additions, from various articles in the "Medical Annual."

Just Published. Crown 8vo. 1/6 net.

**A POCKET BOOK OF CLINICAL METHODS.** By CHAS. H. MELLAND, M.D Lond., M.R.C.P., Phys. Ancoats Hosp.

THIRD EDITION. 12th Thousand. Small 8vo. Illustrated with 200 Original Drawings, 2/6. Lantern Slides of the Illustrations, 1/- and 1/6 each.

**"FIRST AID" TO THE NJURED AND SICK:** An Advanced Ambulance Handbook. By F. J. WARWICK, B.A., M.B., and A. C. TUNSTALL, M.D., F.R.C.S.

THIRD EDITION. 2/- each, or 27/6 the Set of 16 Sheets, or mounted on linen, 45/- With nickel head.

(Adopted by the War Office, Admiralty, and London School Board.)

**LARGE SHEET "FIRST AID" DIAGRAMS:** Being Enlargements of the Illustrations in the above work on sheets 26 in. by 40 in., suitable for lectures and classes.

Bristol: JOHN WRIGHT & CO.

### MEDICAL ACCOUNTS.

Now is the time to arrange for this troublesome business, and

WRIGHT'S THREE-BOOK SYSTEM,
WRIGHT'S SINGLE-BOOK SYSTEM,
(11th Edition)
WRIGHT'S OBSTETRIC RECORDS,
WRIGHT'S VISITING LISTS, 1904,
WRIGHT'S CHARTS of all kinds,
(Nearly 400,000 of which have been sold)
WRIGHT'S CHART HOLDERS,
(In use all over the world)
WRIGHT'S CERTIFICATE BOOKS,
PROFESSIONAL ACCOUNT FORMS,
LETTER HEADINGS, and
DISPENSING LABELS.

Are most of them better than the ordinary run of these things, and their use saves time and trouble.

PLEASE SEND FOR FREE SAMPLES.

London: SIMPKIN & CO., Ltd.

Second Edition. Crown 8vo., price 3/6 net.
With original "Plea—1900—for the State Registration of Nurses."

**MOCK NURSES OF THE LATEST FASHION.**

By FREDERICK J. GANT, F.R.C.S.,
Consulting Surgeon to the Royal Free Hospital.

BAILLIÈRE, TINDALL & COX,
Henrietta Street, Covent Garden, London.

Crown 8vo. Illustrated. Price 2s. 6d. net.

**ANÆSTHETICS.** By J. BLUMFELD, M.D.Cantab., Senior Anæsthetist to St. George's Hospital; Anæsthetist to the Grosvenor Hospital for Women and Children, London, and to the National Dental and Metropolitan Hospitals.

*Lancet.*—"....In writing this handbook the author has been quite successful." *Australasian Medical Gazette.*—"We can strongly commend this book." *Medical Chronicle.*—"The book is one which we can heartily recommend to senior students and practitioners."

London: BAILLIÈRE, TINDALL & COX, 8 Henrietta St., Covent Garden.

**Publications**—*continued.*

**Published January 1st. Price 2s. 6d.**

THE

### Management of Lateral Curvature of the Spine and Stooping.

AND

### "The Development of the Chest in Phthisis."

WITH NUMEROUS ILLUSTRATIONS.

**By E. NOBLE SMITH, F.R.C.S.Edin.**

Senior Surgeon to the City Orthopædic Hospital London.

Author of The Atlas of Histology (jointly with Dr. Klein); The Surgery of Deformities; Spinal Caries. Second Edition, 5s.; Curvatures of the Spine. Fourth Edition. 5s.; Fractures in the Neighbourhood of Joints, 1s.; Paralytic Deformities of the Lower Extremities, 5s.; Spasmodic Wry-Neck, 2s., &c.

**Published by SMITH, ELDER & CO., 15 Waterloo Place, London.**

### NOTICE TO CORRESPONDENTS.

All MSS., Letters, Books for Review, and other matters intended for the Editor should be addressed The Editor, "The Hospital," The Hospital Buildings, 28 & 29 Southampton Street, Strand, W.C.

The Editor cannot undertake to return rejected MSS., even when accompanied by stamped directed envelope.

**Notice to Advertisers and Subscribers.**

All Advertisements, Orders for Copies of The Hospital, and Business Communications should be addressed to **THE MANAGER (Not to the Editor),** The Hospital, 28 & 29 Southampton Street, Strand, London, W.C.

The Cost of Subscription, post free, is as follows:—Per week, 3½d.; per quarter, 3s. 9d.; per half-year, 7s. 6d.; per annum, 15s. Foreign and Colonial:—Per annum, 19s. 6d., payable, in all cases, in advance.

**NOTICE.—Vol. XXXIV. of "The Hospital" (April 1903 to September 1903), in handsome cloth binding, is now on sale at the office of this Journal, price 10s. 6d. Cases for binding the Numbers contained in this Volume 2s. Index to Vol. XXXIV., 2½d., post free.**

**The Monthly part for February is now ready. Price 1s., by post, 1s. 3d.**

### BOOKS RECEIVED.

P. S. KING AND SON.

"Report of Public Health Committee."

T. B. BROWNE, LTD.

"T. B. Browne's Directory, 1904."

MR. MURRAY.

"A Manual of Pathology." By S. Martin.

VINTON & CO.

"Notes on Alcohol." By Sir W. Gilbey, Bart.

JOHN LONG.

"Nurse Charlotte." By L. T. Meade.

REPRINT FROM MEDICAL PRESS AND CIRCULAR.

"Midwives Act, 1902, Effect."

HIS MAJESTY'S STATIONERY OFFICE.

"Local Government Board Report on Lead Poisoning and Water Supplies.

### The Student of Medicine.

### APPOINTMENTS.

**A. J. H. Boyton**, M.R.C.S., L.R.C.P.Lond., Certifying Factory Surgeon for the Watlington District, County Oxford. **H. Coates**, M.D.Vict., D.P.H., Medical Officer of Health and Medical Superintendent of the Isolation Hospital of the Hornsey Borough Council. **H. R. Lloyd Davies**, M.D.Edin., District Medical Officer of the Cheltenham Union. **D. R. Edwards**, L.R.C.P.&S.Edin., Anæsthetist to the Swansea Hospital. **D. E. Evans**, M.B., B.S.Lond., Medical Officer Out-patients' Department Swansea Hospital. **W. Fowler**, L.S.A., D.P.H., District Medical Officer of the Sunderland Union. **G. R. Green**, L.R.C.P.Edin., M.R.C.S., District Medical Officer of the Ripon Union. **John Hay**, M.D., Ch.B.Vict., M.R.C.S.Eng., M.R.C.P.Lond., Physician to the Liverpool Stanley Hospital. **George Hern**, L.R.C.P., L.R.C.S., L.D.S.Eng., Dental Surgeon to the Royal Dental Hospital of London. **John Highet**, M.D., D.P.H., Medical Officer of Health for Burgh of Prestwick, Ayrshire. **A. H. Hoffman**, M.D St.And., M.R.C.S., District Medical Officer of the Ellesmere Union. **R. H. Martin**, M.B., Ch.B Edin., First Assistant Medical Officer to the Cleveland Street Asylum of the Central London Sick Asylum District. **J. Milne**, M.B., District Medical Officer of the Oldham Union. **F. Victor Milward**, B.A., M.B., B.C Cantab., F.R.C.S.Eng., Surgeon to Out-patients to the Birmingham and Midland Free Hospital for Sick Children. **T. P. Lockhart Mummery**, M.B., B.C.Cantab., F.R.C.S.Eng., Assistant Surgeon to St. Mark's Hospital for Fistula and Other Diseases of the Rectum. **W. P. R. Newth**, L.S.A., District Medical Officer of the Northampton Union. **Sidney Herbert Perry**, M.D.Lond., M.B., Ch.B.Birm., M.R.C.P., Physician to Out-patients at the Birmingham and Midland Hospital for Sick Children. **James Phillips**, F.R.C.S.Edin., Honorary Surgeon to the Bradford (Yorks) Children's Hospital. **J. Scott Riddell**, C.M.etc., Aberd., Additional Examiner in Clinical Surgery, Edinburgh University. **H. M. Stumbles**, M.B., Ch.B.Edin., Medical Officer of Health for the A[illegible] Urban District.

### "The Hospital" Institutional Library.

"Hospitals and Asylums of the World." 4 Vols., with a Portfolio of Plans, complete £12 12s.

"Burdett's Hospitals and Charities, 1903." 5s. net.

"Burdett's Official Nursing Directory, 1903." 3s. net.

"Cottage Hospitals, General, Fever, and Convalescent." 10s. 6d.

"Uniform System of Accounts for Hospitals and Public Institutions." New Edition. 4s. net.

"Hospital Expenditure: The Commissariat." 2s. 6d.

"A Legal Handbook for the Use of Hospital Authorities." 2s. 6d.

"The Cottage Hospital Case Book or Register of Patients." 10s. and 12s. 6d.

All these are published by the Scientific Press, Ltd., and may be obtained through any bookseller, or direct from the publishers, 28 and 29 Southampton Street, Strand, London, W.C.

### Hospital Matrons,

Do you know the best book on how to provide for the Hospital Household? In

### HOSPITAL EXPENDITURE: The Commissariat,

Price **2/6** post free, you will find every question considered which bears upon this important subject. The varying circumstances of large and small institutions are carefully considered, and from the making of beef-tea to the complete cost of diets everything that can interest and assist an intelligent manager is within its pages.

You can compare your methods with those of other Hospitals, because careful comparisons are made, even in the matter of the sale of waste material. Matrons about to occupy new posts will find this book invaluable, as it will place them at once in that position of power which knowledge gives.

### The Cottage Hospital Matron will find useful

### COTTAGE HOSPITALS,

**By Sir HENRY BURDETT, K.C.B.**

Price **10/6** post free, 3rd Edition, and a little pamphlet on the

### FURNISHING AND APPLIANCES OF A COTTAGE HOSPITAL,

**By the Hon. SYDNEY HOLLAND,**

Price **6d.** post free; and those matrons who have no secretarial assistance, and all secretaries and medical officers will find invaluable

### THE COTTAGE HOSPITAL CASE-BOOK,

**Or, REGISTER OF PATIENTS.**

Price **10/-** post free.

It is arranged for 150 in-patient cases, with rulings for 8 weeks' payments to each case.

Larger size, containing space for about 300 in-patient cases, **12/6** post free.

All these books can be obtained at all Booksellers', and at
THE SCIENTIFIC PRESS, 28 Southampton Street, Strand, W.C.

### Two Important Text-Books.

### ELEMENTARY ANATOMY AND SURGERY FOR NURSES.

By WILLIAM McADAM ECCLES, M.S.Lond., M.B., F.R.C.S., L.R.C.P.

Crown 8vo., profusely illustrated, cloth, **2/6** post free.

"The book will be valuable to nurses who are beginning the study of anatomy. The instruction is given very clearly and concisely, and the reader is able to glean much information in a very condensed form."—*Nursing Notes.*

### ELEMENTARY PHYSIOLOGY FOR NURSES.

By C. F. MARSHALL, M.D., B.Sc., F.R.C.S.

Crown 8vo., illustrated, cloth, **2/-** post free.

"Nurses will find in it all that they require to know of the subjects treated."—*Daily Chronicle.*

*To be obtained of all Booksellers, or of*

THE SCIENTIFIC PRESS, LIMITED,
28 & 29 Southampton Street, Strand, London, W.C.

### WORKS FOR NURSES.

**LESSONS ON MASSAGE.** By Mrs. MARGARET D. PALMER, Manager of the Massage Department and Instructor of Massage to the Nursing Staff of the London Hospital. Second Edition enlarged and revised; pp. xvi. + 262 with 118 Illustrations, plain and coloured. *Just Published.* Price 7/6 net.

**A MANUAL FOR STUDENTS OF MASSAGE.** By Miss M. A. ELLISON, L.O.S. Second Edition, edited by Miss G. MANLEY, pp. xii + 126. With 50 original Illustrations. *Just Published.* Price 3/6 net.

**A MANUAL FOR HOME NURSING.** By BERNARD MYERS, M.D., M.R.C.S., Surgeon and Lecturer to St. John's Ambulance Association. *Just Published.* Price 2/6 net.

**NOTES ON HOME NURSING.** By Miss D. M. GOLDIE, Lecturer on Nursing, Hygiene, &c., to the County Councils of London, Surrey and Middlesex. *Just Published.* Price 1/6 net.

**THE FEEDING AND HYGIENE OF INFANTS AND CHILDREN.** By JOHN McCAW, M.D., M.R.C.P., Physician to the Belfast Hospital for Children. *Just Published.* Price 2/6.

**MIDWIFERY FOR MIDWIVES.** Designed to cover the requirements of the Examining Board authorised by the Midwives Act. With Appendix on Examinations. By W. DENISON WIGGINS, M.R.C.S., L.R.C.P., &c. *Just out.* 3/6 net.

**DISEASES OF THE HAIR, INCLUDING GREYNESS, BALDNESS, &c., AND THEIR TREATMENT.** By DAVID WALSH, M.D., Western Hospital for Diseases of the Skin. Price 2/6 net.

**MENSTRUATION AND ITS DISORDERS.** By ARTHUR E. GILES, M.D., B.Sc., F.R.C.S., Surgeon Chelsea Hospital for Women. Price 2/6 net.

**MOVABLE ATLAS OF THE ANATOMY AND PHYSIOLOGY OF THE FEMALE GENERATIVE ORGANS AND OF PREGNANCY.** Second Edition. With Text. By ARTHUR E. GILES, M.D., F.R.C.S. Price 3/- net.

**MOVABLE ATLAS OF THE ANATOMY AND PHYSIOLOGY OF THE CHILD.** With Explanatory Text. By D'ARCY POWER, M.A.Oxon, M.B., F.R.C.S. Price 3/- net.

London: BAILLIÈRE, TINDALL & COX, 8 Henrietta Street, Covent Garden.

